# Rethinking the tools of the RNA world

**DOI:** 10.7554/eLife.38297

**Published:** 2018-06-15

**Authors:** Antony Crisp, Thomas Carell

**Affiliations:** Center for Integrated Protein ScienceLudwig-Maximilians-Universität MünchenMunichGermany

**Keywords:** ribozyme, RNA, origins of life, molecular evolution, ribosome, triplets, None

## Abstract

An artificially evolved ribozyme can catalyse the synthesis of RNA by using trinucleotide triphosphates as building blocks.

**Related research article** Attwater J, Raguram A, Morgunov AS, Gianni E, Holliger P. 2018. Ribozyme-catalysed RNA synthesis using triplet building blocks. *eLife* **7**:e35255. doi: 10.7554/eLife.35255

If you could travel three to four billion years back in time, you would find yourself in a world inhabited by the first primitive cells. But what if you went even further? According to the ‘RNA World’ hypothesis, biological evolution was preceded by chemical evolution: an era in which the tasks that are currently performed by DNA (the storage of genetic information) and proteins (the catalysis of chemical reactions) are performed by RNA molecules (Crick, 1968; Orgel, 1968). Moreover, if placed under evolutionary pressure, RNA can also evolve and adapt to its surroundings (Mills et al., 1967).

Although it may have taken many millions of years for self-replicating nucleic acids to emerge, we can speed up the process in the laboratory by employing a high-throughput technique called in vitro evolution (Joyce, 2007). First, enormous libraries of different RNA strands are tested for their ability to catalyse a certain chemical reaction or bind to a particular substrate. The strands that 'pass the test' are separated and reverse transcribed. Sequences are then amplified with occasional mutations using a method called PCR, and subsequently evolved through repeating the processes over many rounds.

Techniques such as these have been employed to evolve RNA enzymes known as ribozymes, which are capable of connecting multiple pieces of RNA to generate a longer strand or using single building blocks (called nucleotide triphosphates) to assemble a new strand from an RNA template (Bartel and Szostak, 1993; Johnston et al., 2001). To do their job correctly, ribozymes need to fold into specific three-dimensional shapes. However, in many instances, these forms can hinder the replication of the RNA. Now, in eLife, Philipp Holliger and colleagues at the MRC Laboratory of Molecular Biology – including James Attwater as first author – report an artificially evolved polymerase ribozyme that can overcome this obstacle (Attwater et al., 2018).

The researchers used trinucleotide triphosphates (also known as triplets) as building blocks, rather than the more typical mononucleotide triphosphate building blocks. When fed to the active ribozyme, the triplets worked together to unravel those folded RNA structures that would otherwise have prevented the replication process (Figure 1). Unlike the typical building blocks, the triplets bound to the RNA templates in an ordered manner, essentially pre-organising the templates for replication.

Remarkably, the new ribozyme was able to copy a wide range of complex RNA sequences, including its own catalytic domain. Structural analyses also revealed that it had an unusual heterodimeric structure that comprised of a catalytic portion and a non-catalytic RNA co-factor. Although this kind of dimerization has been seen before in RNA evolution, the spontaneous emergence of two distinct and cooperative RNA molecules had not previously been observed in an active ribozyme (Suslov et al., 2015).

**Figure 1.**
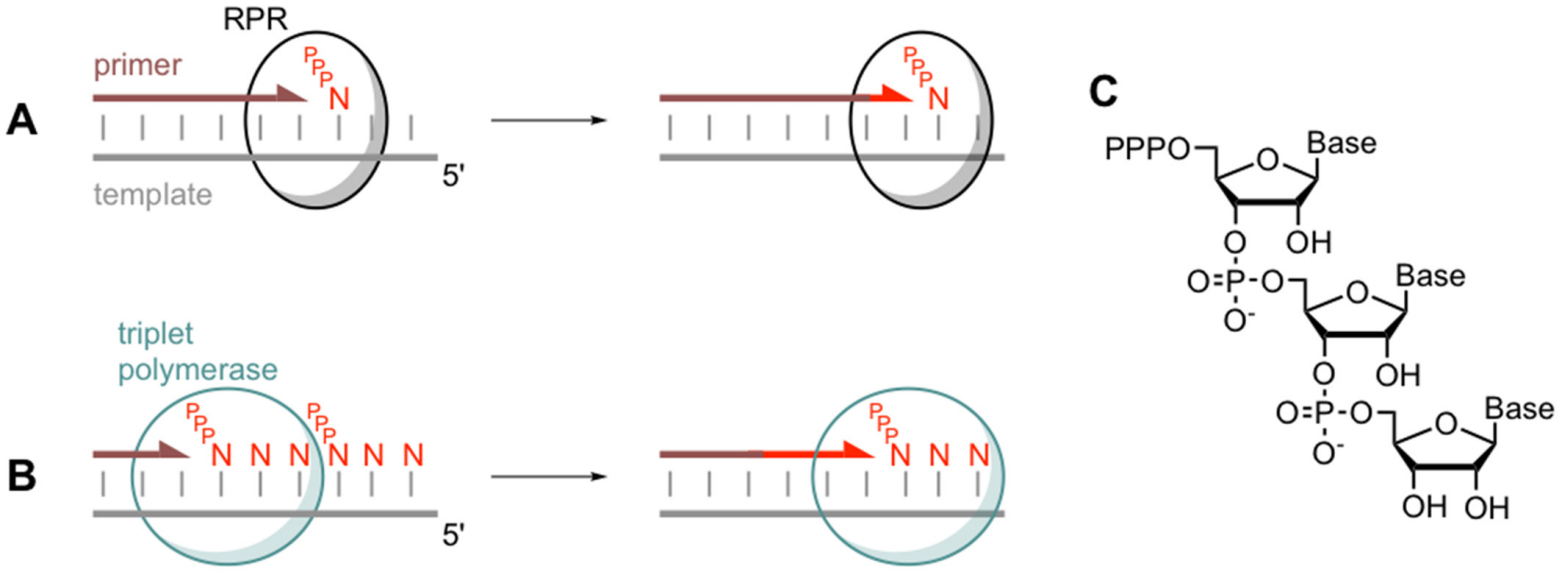
Comparing monomer and triplet polymerisation in RNA-templated primer extension. (**A**) An RNA polymerase ribozyme (RPR; black circle) adds a mononucleotide triphosphate (PPPN) building block to the primer (brown line) in a primer/template duplex (left). This process can be repeated (right). (**B**) The triplet polymerase ribozyme developed by Attwater et al. (blue circle) adds trinucleotide triphosphate (PPPNNN) building blocks and has several advantages: for example, it does not always require a primer and can copy RNA sequences in both directions. (**C**) The chemical structure of a trinucleotide triphosphates building block, showing the three nucleotide bases.

The use of triplet building blocks had other advantages: it was possible to copy RNA sequences in both directions (that is, 5’ – 3’ and 3’ – 5’), and copying could be initiated anywhere along a template, sometimes even without the use of a primer (Figure 1). According to Attwater et al., these emergent properties might have been critical for early RNA machines, given that an ancient enzyme would have needed to be robust and to perform its role as independently as possible.

Many challenges will have to be overcome to demonstrate that RNA, by itself, could have supported an evolving genetic system. If RNA were to have existed as a sole genetic biopolymer, it would have needed to be able to replicate itself efficiently, completely, and without the help of proteins. Although evidence for an RNA-copying machinery is likely to be found within a small ribosomal subunit, an ancient ribozyme with intact RNA replication activity has yet to be discovered (Weiss and Cherry, 1993). Ribozymes like those developed by Attwater et al. are nonetheless starting to blur the distinction between chemicals and true living things. It will be exciting to see how the field develops in coming years as we move closer to being able to demonstrate a truly self-sustaining and evolvable genetic polymer in the lab.
